# Comparison of clinical outcomes in patients with acute ischemic stroke who underwent endovascular treatment using different perfusion modalities: a real-world multicenter study

**DOI:** 10.3389/fneur.2023.1275715

**Published:** 2023-10-25

**Authors:** Jiali Gao, Zhen Jing, Shengming Huang, Jiajie Yang, Min Guan, Shijun Zhang, Hao Li, Yongxin Li, Kui Lu, Ming Yang, Li’an Huang

**Affiliations:** ^1^Department of Neurology, Clinical Neuroscience Institute, First Affiliated Hospital of Jinan University, Guangzhou, China; ^2^Department of Neurology, The Fourth Affiliated Hospital of Guangzhou Medical University, Guangzhou, China; ^3^Department of Neurology, Maoming People’s Hospital, Maoming, China; ^4^Department of Neurology, Shunde Hospital of Southern Medical University, Foshan, China; ^5^Department of Neurology, Zhongshan People’s Hospital, Zhongshan, China; ^6^Neuroblem Limited Company, Shanghai, China

**Keywords:** computed tomography perfusion, arterial spin labeling, stroke, endovascular treatment, imaging

## Abstract

**Background:**

Advanced perfusion modalities are increasingly popular for various diseases. However, few studies have focused on contrasting perfusion patterns.

**Objective:**

This study aimed to compare the time efficiency and clinical outcomes of patients with acute ischemic stroke (AIS) who underwent endovascular treatment (EVT) before one-stop arterial spin labeling (ASL) and computed tomography perfusion (CTP) protocols.

**Methods:**

This study retrospectively included 326 patients with AIS who had accepted EVT within 24 h of onset from four comprehensive stroke centers between October 2017 and September 2022. After 1:1 matching of the propensity scores, 202 patients were separated into two groups: the ASL group (*n* = 101) and the CTP group (*n* = 101).

**Results:**

Functional independence at 90 days (modified Rankin Scale [mRS] 0–2; *p* = 0.574), onset-to-puncture time (*p* = 0.231), door-to-puncture time (*p* = 0.136), and door-to-perfusion time (*p* = 0.646) were not significantly different between the two groups. The proportion of EVT complications (31.7% in the ASL group vs. 14.9% in the CTP group, *p* = 0.005) and symptomatic intracranial hemorrhage (sICH) at 24 h (23.8% in the ASL group vs. 9.9% in the CTP group, *p* = 0.008) in the CTP group were lower than the ASL group. The ischemic core volume was a common predictor of favorable outcomes in both ASL (*p* < 0.001) and CTP (p < 0.001) groups.

**Conclusion:**

There were no significant differences in time efficiency and efficacy outcomes between the two groups of patients receiving one-stop ASL and CTP. The proportion of sICH at 24 h and EVT complications of patients in the CTP group was lower than the ASL group. The ischemic core volume was an independent predictor for favorable outcomes.

## 1. Introduction

Endovascular treatment (EVT) is an effective and safe treatment for acute ischemic stroke (AIS) (1, 2). Advanced perfusion modalities are increasingly used for selecting patients to undergo EVT (3–5). These modalities are able to quantify the volume of ischemic penumbra, core infarct, and hypoperfusion (6–9). EVT is important for salvaging the recoverable ischemic penumbra, which improves the functional prognosis of patients with AIS (10, 11).

Several studies have investigated pre-interventional imaging and found that the clinical prognosis after EVT was better in patients selected for perfusion imaging than in those without it (12, 13). The DEFUSE-3 and DAWN trials demonstrated that advanced perfusion imaging could be used to assist in selecting patients for EVT (14, 15). Based on the results from two trials above, stroke guidelines in the United States and Europe recommend the use of advanced perfusion for EVT (16, 17). One-stop arterial spin labeling (ASL) and one-stop computed tomography perfusion (CTP) are the two main advanced perfusion imaging modalities (18); however, it is unclear which perfusion modality is more helpful in patient selection.

Therefore, this study aimed to retrospectively compare the time efficiency, efficacy outcomes, and safety outcomes of patients with AIS who received one-stop ASL and one-stop CTP before EVT within 24 h of onset using multicenter data.

## 2. Methods

### 2.1. Ethics

This study was endorsed by the Medical Ethics Committee of the First Affiliated Hospital of Jinan University, and all patients signed an institutional informed consent form.

### 2.2. Patients

**Figure 1:**
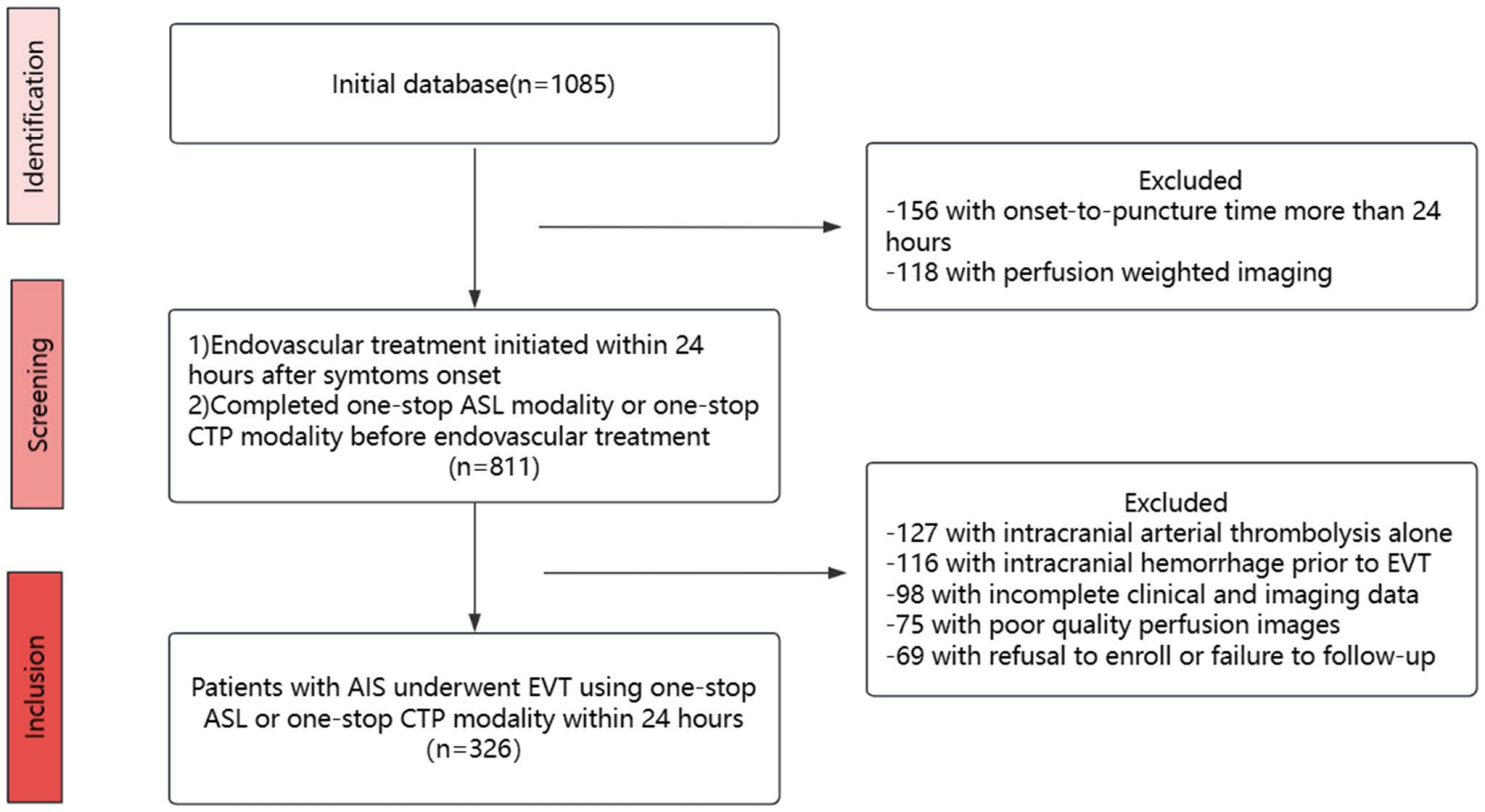
Flow chart. EVT, endovascular treatment; ASL, arterial spin labeling; CTP, computed tomography perfusion.

The study collected participants who were enrolled in four Guangdong district general stroke centers within 24 h of symptoms of AIS beginning to appear between October 2017 and September 2022. The data were obtained from a multicenter prospective registry, the Multi-center Prospective Registry Study for Endovascular Treatment of Acute Ischemic Stroke (ChiCTR 1900028224). The inclusion criteria were as follows: (1) patients aged 18 years or older; (2) patients who were treated with emergency EVT within 24 h of onset; (3) patients who completed one-stop ASL or CTP modality before EVT. The following criteria were used to exclude candidates: (1) intracranial arterial thrombolysis alone, (2) intracranial hemorrhage prior to EVT, (3) incomplete clinical and imaging data, (4) poor quality perfusion images, and (5) refusal to enroll or failure to follow-up. After the inclusion and exclusion of patients, there were 225 in the ASL group and 101 in the CTP group, of whom 105 were from a multicenter prospective study and 221 from a single-center retrospective study (selection details are presented in Figure 1); however, both groups had an imbalanced baseline. Therefore, we applied a 1:1 matching propensity score to reduce the effects of confounding factors (matching details are shown in Table 1). After matching, 101 patients were included in the ASL group and 101 patients in the CTP group. Patients were selected for EVT primarily based on the National Institutes of Health Stroke Scale (NIHSS) scores, Alberta Stroke Program Early CT Score (ASPECTS), and perfusion mismatch on CT or magnetic resonance imaging by visual inspection of neurointerventionalists and neurologists. In the prior EVT, all patients were assigned to two groups: ASL and CTP.

**Table 1:** Baseline characteristics of patients between ASL and CTP before and after propensity score matching.

	Before propensity score matching			After propensity score matching		
	ASL (*n* = 225)	CTP (*n* = 101)	*p*-value	ASL (*n* = 101)	CTP (*n* = 101)	*p*-value
*Demographics*						
Age, median (IQR)	63 (56–73)	70 (58–77)	0.004	66 (60–75)	70 (58–77)	0.285
Male sex, *n* (%)	151 (67.19)	69 (68.3)	0.830	73 (72.3)	69 (68.3)	0.538
*Medical history*						
Smoking, *n* (%)	56 (25)	20 (19.8)	0.306	27 (26.7)	20 (19.8)	0.244
Drinking, *n* (%)	26 (13.5)	9 (9)	0.257	9 (8.9)	9 (8.9)	>0.999
Hypertension, *n* (%)	128 (56.9)	68 (67.3)	0.075	64 (63.4)	68 (67.3)	0.554
Diabetes, n (%)	69 (30.7)	39 (38.6)	0.159	37 (36.6)	39 (38.6)	0.771
Hyperlipidemia, n (%)	104 (54.2)	83 (83)	<0.001	86 (85.1)	84 (83.2)	0.700
Atrial fibrillation, n (%)	52 (23.2)	32 (31.7)	0.107	18 (18.0)	32 (31.7)	0.025
Acute coronary syndrome, *n* (%)	43 (19.1)	16 (15.8)	0.478	19 (18.8)	16 (15.8)	0.577
Valvulopathy, *n* (%)	15 (6.7)	9 (8.9)	0.473	4 (4)	9 (8.9)	0.251
Ischemic stroke, *n* (%)	43 (19.1)	18 (17.8)	0.783	19 (18.8)	18 (17.8)	0.856
*Admission clinical data*						
SBP, median (IQR)	137 (123–154)	132 (118–145)	0.052	143 (125–158)	132 (118–145)	0.005
DBP, mean (SD)	80 (16)	74 (16)	0.003	81 (16)	74 (16)	0.002
FBG, median (IQR)	6.5 (5.7–8.2)	7.2 (6.2–8.8)	0.001	6.7 (5.6–8.3)	7.2 (6.2–8.8)	0.022
Baseline NIHSS, median (IQR)	13 (9–17)	16 (9–21)	0.003	15 (11–19)	16 (9–21)	0.355
Baseline ASPECTS, median (IQR)	7 (6–9)	7 (6–8)	0.048	7 (5–8)	7 (6–8)	0.477
HDL cholesterol, median (IQR)	0.97 (0.81–1.62)	1.05 (0.85–1.20)	0.049	1.01 (0.87–1.11)	1.04 (0.86–1.20)	0.112
LDL cholesterol, mean (SD)	2.62 (1.98–3.34)	2.53 (2.00–3.14)	0.245	2.84 (0.92)	2.60 (0.90)	0.058
HbA1c, median (IQR)	6 (5.5–6.6)	6 (5.5–6.9)	0.698	6.1 (5.7–6.5)	6.1 (5.6–6.8)	0.826
*Occlusion vessel, n (%)*			0.785			0.628
ICA	60 (26.7)	22 (25.2)		29 (28.7)	22 (21.8)	
MCA	110 (48.9)	50 (49.5)		42 (41.6)	50 (49.5)	
ICA + MCA	16 (7.1)	10 (9.9)		10 (9.9)	10 (9.9)	
ACA	6 (2.7)	4 (4.0)		2 (2.0)	4 (4.0)	
BA	33 (14.7)	15 (14.9)		18 (17.8)	15 (14.9)	
*TOAST classification, n (%)*			0.191			0.560
LAA	139 (61.8)	72 (71.3)		71 (70.3)	72 (71.3)	
CE	71 (31.6)	22 (21.8)		26 (25.7)	22 (21.8)	
Others/unknown	15 (6.7)	7 (6.9)		4 (4.0)	7 (6.9)	
*Treatment, n (%)*			0.647			0.831
Direct EVT	200 (88.9)	88 (87.1)		89 (88.1)	88 (87.1)	
IVT + EVT	26.2 (11.1)	13 (12.9)		12 (11.9)	13 (12.9)	
Reperfusion after EVT, n(%)	207 (92.4)	96 (95)	0.381	93 (92.1)	96 (95.0)	0.390
Hypoperfusion, volume (ml), median (IQR)	69.1 (31.5–124.6)	115.8 (83.4–198.3)	<0.001	68.4 (35.2–120.55)	114.2 (80.5–194.9)	<0.001
Ischemic core, volume (ml), median (IQR)	15.1 (9.8–30.8)	37.4 (16.9–60.3)	<0.001	14.6 (9.6–28.5)	36.5 (14.5–55.5)	<0.001
Mismatch ratio, median (IQR)	4.3 (3.4–5.1)	4.1 (2.8–5.4)	0.084	4.5 (3.5–5.0)	3.9 (2.6–5.3)	0.076

IQR, interquartile range; ASL, arterial spin labeling; CTP, computed tomography perfusion; SBP, systolic blood pressure; DBP, diastolic blood pressure; ICA, internal carotid artery; MCA, middle cerebral artery; ACA, anterior cerebral artery; BA, basilar artery; FBG, fasting blood glucose; NIHSS, National Institutes of Health Stroke Scale; ASPECTS, Alberta Stroke Program Early CT Score; LAA, large artery atherosclerosis; CE, cardiogenic; EVT, endovascular treatment; IVT, intravenous thrombolysis.

Demographic data and clinical information of the patients were collected. Based on the ORG 10172 Trial in Acute Stroke Treatment Classification, stroke etiology can be divided into three categories: large atherosclerosis, cardioembolism, and others/unspecified (19). The occlusion site of the vessel was classified as ICA, MCA, ACA, BA, or tandem occlusion. Tandem occlusion was defined as arterial occlusion in two or more different parts of a continuous vessel (20). The modified thrombolysis on the cerebral infarction (mTICI) scale assessed reperfusion status after the neurointervention: 0-2a and 2b-3, graded according to whether 50% of the affected vascular territory was re-perfused (21).

### 2.3. ASL protocol

MR examinations were performed using a 1.5 T (GE Healthcare, Milwaukee, WI, USA). Resting-state perfusion imaging was acquired using a pCASL sequence with 3D fast spin-echo acquisition and background suppression. Acquisition parameters were as follows: repetition time (TR) = 4,632 ms, echo time (TE) = 10.5 ms, post-labeling delay = 1.525 s, field of view (FOV) = 240 × 240 mm, slice thickness/slice spacing = 4.0/0 mm, number of slices = 36, and excitation number. The rs-fMRI data were acquired using a gradient-echo planar imaging sequence with the following parameters: TR/TE = 2000/25 ms, FOV = 240 × 240 mm, scan matrix = 64 × 64, flip angle = 90°, excitation number = 1, voxel size = 3.75 × 3.75 × 3 mm^3^, slice thickness/spacing = 3.0/1.0 mm, number of slices = 35, and scan time = 7 min, and a total of 210 time points were scanned. In addition, structural data were acquired using a 3D brain volume imaging sequence covering the entire brain with TR/TE = 8.2/3.2 ms, flip angle = 12°, bandwidth = 31.25 Hz, slice thickness/slice spacing = 1.0/0 mm, scan matrix = 256 × 256, FOV = 240 × 240 mm, number of excitations = 1, and scan time = 3 min 45 s.

### 2.4. CTP protocol

CT was carried out with a 320-slice multidetector CT machine (AquilionOne; Canon Medical Systems, Tokyo, Japan). In this study, 50 mL of the contrast medium containing 370 mg of iodine per mL (Ultravist 370; Bayer, Leverkusen, Germany) was injected at a flow rate of 6 mL/s. The parameters of the CT scan were as follows: tube voltage 80 kV; matrix 512 × 512; field of view 320 mm; rotation time 0.35 s; and collimator 0.5 mm x 320. Nineteen whole-brain scans were acquired for each patient. The scans were loaded into the Vitrea Fx 6.3 workstation (Vital Images, Minnetonka, MN).

### 2.5. Image analysis

**Figure 2:**
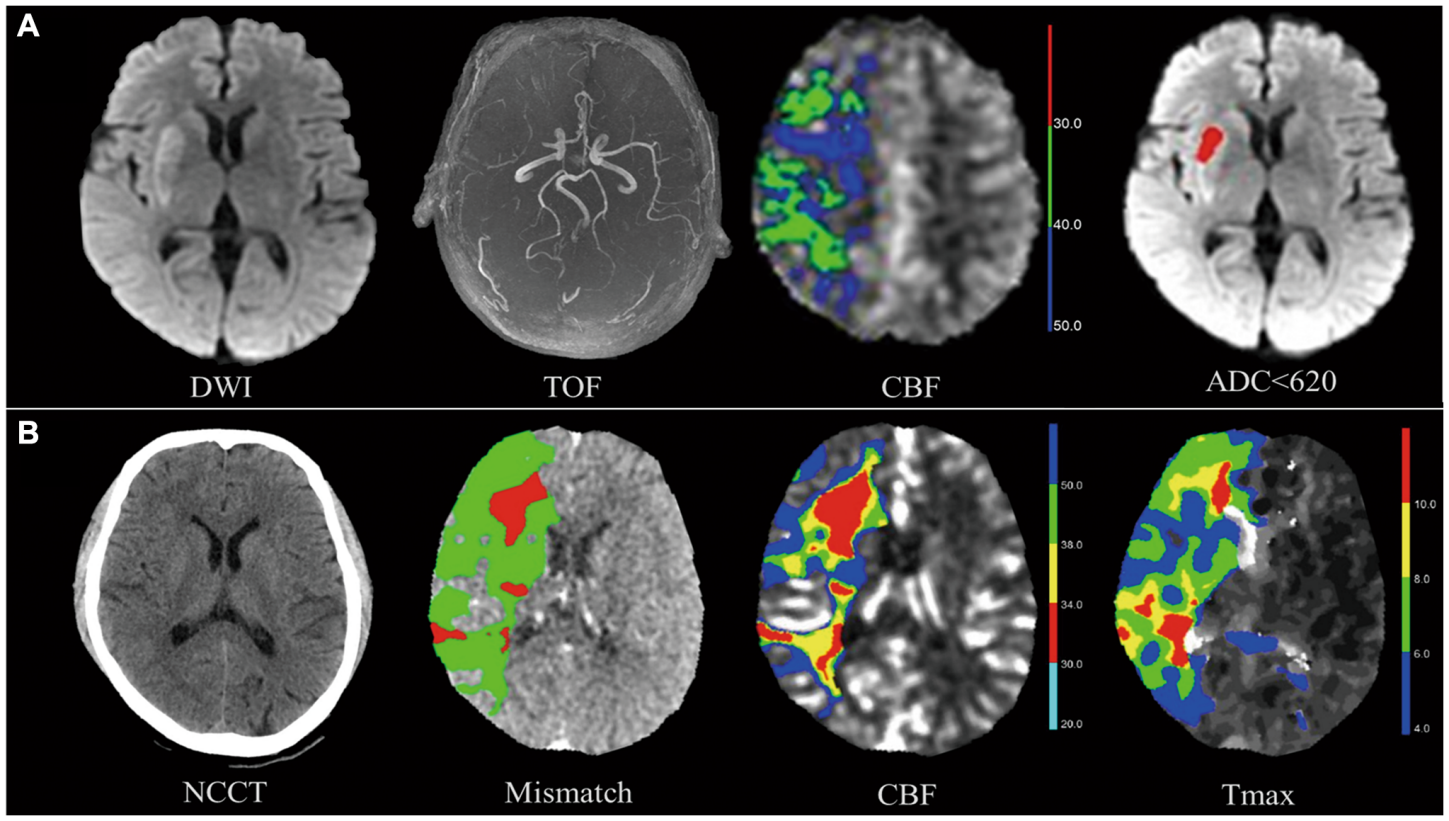
Hypoperfusion volume and ischemic core volume in arterial spin labeling (ASL) and computed tomography perfusion (CTP). The images above show two patients who suffered a right M1-MCA occlusion and neurological deficit (patient A admission NIHSS = 7, patient B admission NIHSS =12) and were successfully treated with thrombectomy (patient A mTICI 2C, patient B mTICI 3). **(A)** Top row shows patient A’s images. On TOF-MRA, DWI shows occlusion of right MCA with infarction of the basal ganglia. Ischemic core volume in ASL, measured by apparent diffusion coefficient (ADC), was 5.7 mL. The volume of ischemic core in ASL defined by apparent diffusion coefficient (ADC) < 620 was 5.7 mL. **(B)** Bottom row shows patient B’s images. The ischemic core volume in CTP defined by CBF < 30% (red) was 45.9 mL. A hypoperfusion volume of 285.1 mL was determined by the time to maximum (Tmax) >6 s (green). As defined by Tmax >6 s (green) - CBF < 30% (red), a mismatch volume of 239.2 mL has been observed. Both patients had excellent neurological recovery at discharge (NIHSS = 1).

The evaluation of CTP and ASL images was performed using F-STROKE (version 1.0.9). The one-stop CTP protocol included computed tomography, multi-period computed tomography angiography (CTA), and CTP. As part of the one-stop ASL protocol, diffusion-weighted imaging (DWI), fluid-attenuated inversion recovery (FLAIR), and MR angiography (MRA) of the head and neck were also included. To define the volume of the ischemic core, the apparent diffusion coefficient (ADC) was multiplied by 620, and the volume of hypoperfusion was defined as cerebral blood flow (CBF) < 40% on ASL (22). The time to maximum (Tmax) > 6 s volume represents hypoperfusion, and a CBF < 30% of volume represents the ischemic core in CTP (23, 24). Details of the hypoperfusion and ischemic core images are illustrated in Figure 2.

### 2.6. Outcome measures

Our clinical outcomes included efficacy and safety. The primary outcome was functional independence (defined as a modified Rankin Scale [mRS] score 0–2) of efficacy outcomes. Efficacy outcomes were mRS score 0–2 at 90 days, NIHSS at discharge, early neurological improvement (ENI), early neurological deterioration (END), and futile recanalization. ENI was defined as a decline in NIHSS score from admission to 24 h ≥ 4 or NIHSS score at 24 h was 0–1 (25). END was an increase in 24-h NIHSS (> 4) from baseline (25). Futile recanalization was discharged with an mRS of 4–6 or deteriorating pre-stroke disability (mRS of 4–6) despite reperfusion success (25). Symptomatic intracranial hemorrhage (sICH) within 24 h, 90-day mortality, and complications associated with EVT were the safety outcomes. sICH at 24 h post-admission was defined as any hemorrhage combined with an increase in the NIHSS score, at least 4 points on any NIHSS score or by at least 2 points on any NIHSS score category following the Heidelberg Classification Scheme (18). Complications related to intervention were hemorrhagic transformation, embolism, arterial dissection, and vasospasm.

### 2.7. Statistical analysis

Propensity score matching was used to minimize the effect of confounders. Our data were primarily analyzed in two separate groups, ASL and CTP. Measured variables are expressed as means or medians using Student’s t-tests and Mann–Whitney U-tests, and qualitative variables are expressed as percentages using Mann–Whitney U-tests and analysis of variance. With the dichotomous variable of outcome as the dependent variable and the observed factors as the independent variables, we used multifactorial logistic regression analysis to derive independent risk factors for outcome. Statistical significance was set at a *p*-value of <0.05. All statistical analyses were performed using SPSS Statistics 27.0 (IBM, Chicago, IL, United States) and R (version 4.2.0, R Foundation for Statistical Computing, Vienna, Austria).

## 3. Results

### 3.1. Patients

There were 101 patients each in the ASL and CTP groups. The median age of the ASL group was 66 years (IQR, 60–75 years), and the median age of the CTP group was 70 years (IQR, 58–77 years), with no statistically significant differences between the two groups (*p* = 0.285). The middle cerebral artery was the most commonly occluded site (41.6% in the ASL group and 50% in the CTP group; *p* = 0.628). The proportion of successful reperfusion in the ASL and CTP groups was 92.1 and 95%, respectively. No significant differences were observed (*p* = 0.390).

Admission systolic blood pressure in the ASL group (median 143, IQR 125–158) and diastolic blood pressure (mean 81, SD 16) were substantially higher than those in the CTP group (median 132, IQR 118–145, *p* = 0.005; mean 74, SD 16, *p* = 0.002, respectively). Meanwhile, fasting blood glucose at admission (median 6.7, IQR 5.6–8.3, ASL group vs. median 7.2, IQR 6.2–8.8, CTP group; *p* = 0.022), the proportion of atrial fibrillation history (18 [18%], ASL group vs. 32 [31.7%], CTP group; *p* < 0.001), hypoperfusion volume (median 68.4, IQR 35.2–120.55, ASL group vs. median 114.2, IQR 80.5–194.9, CTP group; *p* < 0.001), and ischemic core volume (median 14.6, IQR 9.6–28.5, ASL group vs. median 36.5, IQR 14.5–55.5, CTP group; p < 0.001) were higher in the CTP group. Differences were considered statistically significant. Additionally, neither group showed statistically significant differences in other baseline features. The details of demographic, clinical, and neuroimaging data are summarized in Table 1.

### 3.2. Time windows

From onset to puncture, the median time in the ASL group was 383 min (IQR, 245–583 min), whereas it was 333 min in the CTP group, (IQR, 208–486 min). Accordingly, the average door-to-puncture (DPT) time in the ASL group was 78 min (IQR, 53–121 min) compared to 95 min in the CTP group (IQR, 60–127 min). Median door-to-reperfusion time was 138 min for CTP (IQR, 90–206 min) and 97 min for ASL (IQR, 132–181). No significant differences were found in onset-to-puncture time (*p* = 0.231), door-to-puncture time (*p* = 0.136), and door-to-reperfusion time (*p* = 0.646).

### 3.3. Efficacy outcomes

**Figure 3:**
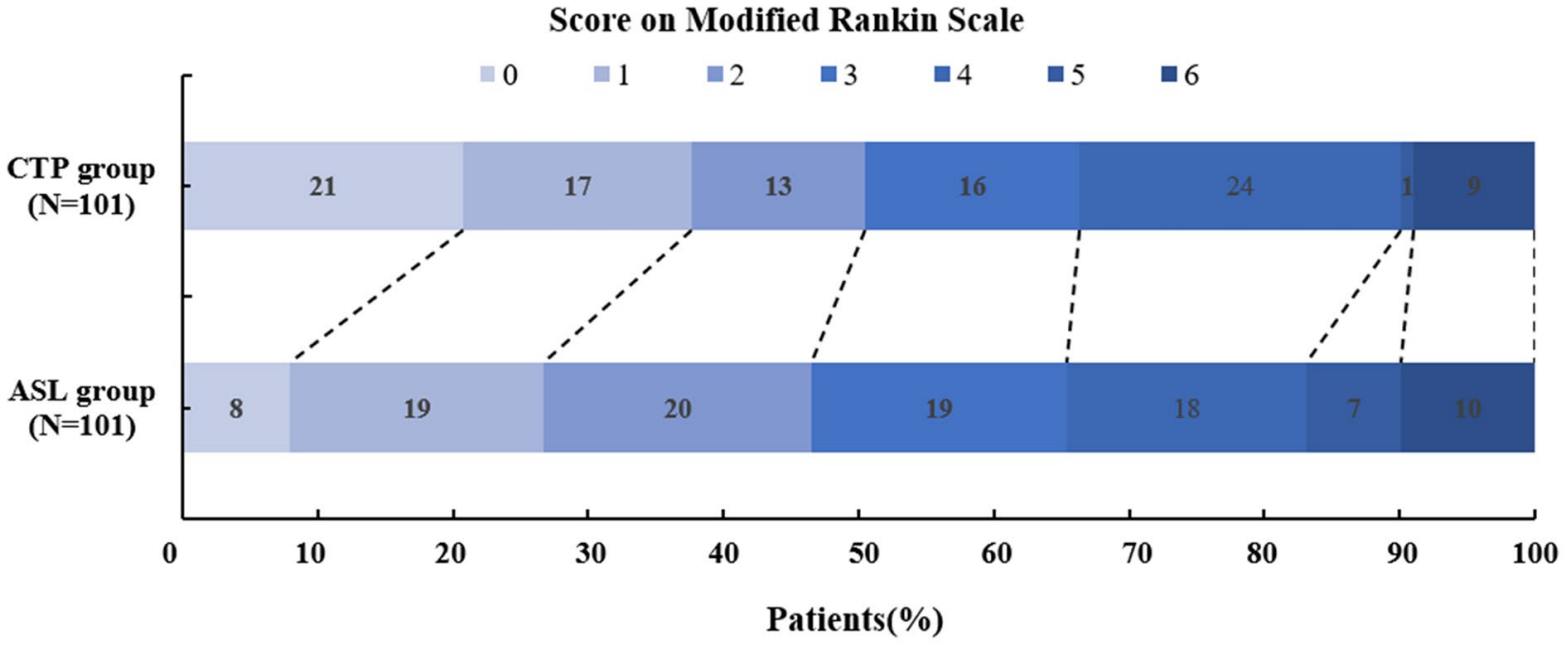
Modified Rankin scale scores at 90 days.

The mRS scores at 90 days (*p* = 0.574), the NIHSS scores at discharge (*p* = 0.670), ENI (*p* = 0.213), END (*p* = 0.149), and futile recanalization (*p* = 0.538) did not display significant differences between the two groups. As shown in Table 2; Figure 3, 47 patients in the ASL group and 51 patients in the CTP group experienced positive outcomes (mRS 0–2). In total, 46.3% of ASL patients had favorable outcomes compared to 50.5% of the CTP patients (*p* = 0.574). ASL-before-EVT patients did not display a significant difference in efficacy outcomes compared with CTP-before-EVT patients.

**Table 2:** Time windows and clinical outcomes after propensity score matching.

	Overall (*n* = 202)	ASL (*n* = 101)	CTP (*n* = 101)	*p*-value
*Time windows*				
OPT, median (IQR)	359 (240–551)	383 (245–583)	333 (208–486)	0.231
DPT, median (IQR)	89 (59–123)	78 (53–121)	95 (60–127)	0.136
DRT, median (IQR)	137 (92–189)	138 (90–206)	97 (132–181)	0.646
*Clinical outcomes, n (%)*				
*Efficacy outcomes*				
mRS 0–2 at 90 days	104 (51.5)	47 (46.5)	51 (50.5)	0.574
NIHSS at discharge, median (IQR)	6 (2–13)	6 (3–13)	6 (2–14)	0.670
ENI	58 (28.7)	25 (24.8)	33 (32.7)	0.213
END	8 (4.0)	6 (5.9)	2 (2.0)	0.149
Futile recanalization	60 (29.7)	28 (27.7)	32 (31.7)	0.538
*Safety outcomes*				
sICH at 24 h	34 (16.8)	24 (23.8)	10 (9.9)	0.008
Complications of EVT	47 (23.3)	32 (31.7)	15 (14.9)	0.005
Mortality at 90 days	19 (9.4)	10 (9.9)	9 (8.9)	0.810

IQR, interquartile range; ASL, arterial spin labeling; CTP, computed tomography perfusion; OPT, onset-to-puncture time; DPT, door-to-puncture time; DRT, door-to-reperfusion time; NIHSS, National Institutes of Health Stroke Scale; sICH, symptomatic intracranial hemorrhage; mRS, modified Rankin Scale; EVT, endovascular treatment.

### 3.4. Safety outcomes

There were 24 sICH cases in less than 24 h in the ASL group and 10 in the CTP group, substantially different between both groups (ASL group, 23.8% vs. CTP group, 9.9%, *p* = 0.008). All-cause death was similar (9.9% for ASL and 8.9% for CTP, *p* = 0.810). There were 32 (7%) and 15 (14.9%) EVT-related events for ASL and CTP, respectively, and a statistically significant difference was found between the two groups (*p* = 0.005). Details of the time windows and outcomes are summarized in Table 2**.**

### 3.5. Multivariable regression analysis for predicting favorable outcomes (mRS 0–2 at 90 days) in the ASL group and CTP group

**Figure 4:**
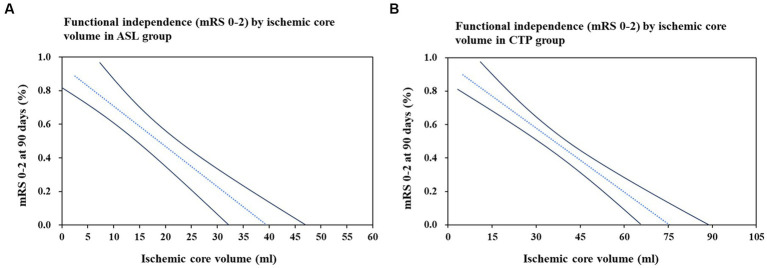
Association between ischemic core volume and functional independence (mRS 0–2) at 90 days. Relationship between ischemic core volume and the functional independence (mRS, modified Rankin Scale 0–2) was assessed using logistic regression models. **(A)** Represents the ASL group and **(B)** is for the CTP group. The dash line is point estimate, and solid lines are 95% confidence interval. Models adjusted for age, Alberta Stroke Program Early CT Score (ASPECTS), ischemic core volume, and mismatch ratio.

To predict favorable outcomes, 101 patients from the ASL group and 101 patients from the CTP group were entered into models with logistic regression for multiple variables. Multivariate logistic regression in the ASL group indicated that hypoperfusion volume (OR: 0.924; *p* = 0.043) and ischemic core volume (OR: 2.752; *p* = 0.002) predicted favorable outcomes at 90 days. Multivariate logistic regression analysis in the CTP group showed that ischemic core volume (OR: 1.220; *p* = 0.028) predicted favorable outcomes. Ischemic core volume was a common predictor in both groups. The ASL and CTP group multivariate regression analyses for the prediction of a favorable outcome at 90 days are shown in Table 3**.** We found that the larger the ischemic core infarct size, the lower the percentage of functional independence in the ASL and CTP groups when analyzing the relationship between ischemic core volume and functional independence. The details of the relationship are shown in Figure 4.

**Table 3:** Multivariable regression analysis for predicting favorable outcome (mRS 0–2 at 90 days) in the ASL group and CTP group after propensity score matching.

ASL (*n* = 101)			CTP (*n* = 101)		
Predictors	OR [95%CI]	*p*-value	Predictors	OR [95%CI]	*p*-value
Age	1.011 (0.935–1.093)	0.784	Age	1.024 (0.957–1.096)	0.490
ASPECTS	0.937 (0.514–1.707)	0.832	ASPECTS	0.875 (0.546–1.402)	0.578
Hypoperfusion volume	0.921 (0.850–0.998)	0.043	Hypoperfusion volume	1.004 (0.969–1.040)	0.840
Ischemic core volume	2.752 (2.028–4.477)	0.002	Ischemic core volume	1.220 (1.021–1.457)	0.028
Mismatch ratio	4.450 (1.586–12.489)	0.005	Mismatch ratio	0.936 (0.329–2.659)	0.901

mRS, modified Rankin Scale; ASPECTS, Alberta Stroke Program Early CT Score; ASL, arterial spin labeling; CTP, computed tomography perfusion.

## 4. Discussion

Given the limited studies comparing perfusion modalities, we aimed to compare the ASL and CTP imaging modalities. This study reported no significant difference for 90-day favorable functional outcomes and time efficiency between the ASL and CTP groups. However, the proportion of sICH and EVT complications in the CTP group was significantly lower than the ASL group. The ischemic core volume was found to be a reliable predictor for favorable prognosis.

Our results did not show a statistically significant difference between the ASL and CTP groups in the advanced perfusion imaging the onset-to-puncture time, door-to-puncture time, and door-to-reperfusion time. Thus, there are no significant differences in efficacy outcomes, suggesting that both pre-intervention options are feasible in terms of time and efficacy outcomes. However, both modalities have their advantages and disadvantages. Compared with ASL, one-stop CTP has a shorter scanning time, broader adaptation to the population, higher clarity of vascular imaging, and higher accuracy of perfusion imaging. Therefore, the initial assumption of our study was that the door-to-puncture time or door-to-reperfusion time of the CTP group would be significantly shorter than that of the ASL group. However, the results were inconsistent with the assumption. The possible explanation may be that for participants in the CTP group who came to the hospital outside normal working hours, the duty nurse in the imaging department spent time rushing from home to the hospital to provide the patients with a contrast injection, which increases the waiting time. Thus, patients undergoing CTP were admitted later than those undergoing ASL patients; most patients were admitted during the COVID period. Upon arrival at the hospital, patients had to undergo complete viral nucleic acid testing and provide a negative viral nucleic acid before receiving further perfusion imaging and vascular interventions, a complex process that consumed more time. Therefore, future studies will hopefully compare the temporal differences between the two perfusion modalities over the same period.

Although our results did not find a correlation between door-to-puncture time and outcomes, it was found that ischemic core volume analyzed by perfusion imaging was strongly associated with favorable clinical outcomes. Furthermore, in a multivariable logistic regression analysis, we found that ischemic core volume was a reliable factor of primary clinical prognosis. Smaller infarct core volume was related to patients with more favorable functional independence, more effective recanalization, a smaller proportion of sICH at 24 h, and fewer postoperative complications. Thus, infarct volume possibly was an independent predictor of clinical outcomes, not time efficiency, which aids in selecting EVT patients.

Moreover, our results did not find a difference in time efficiency. Nevertheless, the sICH at 24 h, EVT complications, hypoperfusion volume, and ischemic core volume were significantly different. We analyzed the reasons for the differences in the safety outcomes as follows. First, as a contrast-based imaging modality, one-stop CTP had better vessel definition and fewer artifacts compared to one-stop ASL. The interventionalist receives more accurate information, such as the location and characteristics of the thrombus prior to EVT. As a result, they are likely to select a more appropriate intervention, potentially reducing complications and sICH of EVT. Second, with the iterative updating of embolization devices and the increasing skill of neurointerventionists, EVT complications such as vessel injury leading to bleeding or thrombus escape causing distal vessel embolization have become less likely. Third, with the increased awareness of ischemic stroke, the ability to detect stroke has increased, and onset-to-puncture time has decreased. In patients with a shorter onset time, the interventionist may have more time to preserve a more significant number of ischemic penumbrae, and the patient may ultimately have a smaller core infarct size. These may reduce the rate of infarct hemorrhagic transformation or even sICH after revascularization. Although the choice of both perfusion modalities before EVT is acceptable, individualization based on the condition is most reasonable.

## 5. Limitations

There were several limitations to this study. First, this was a multicenter retrospective study of patients treated with EVT only, which may limit the generalizability of the study, and the quality of a retrospective study might be uneven. However, the data were from a registry multicenter cohort study, and we applied a propensity match score to reduce confounding factors. Second, we only compared the ASL and CTP groups rather than the perfusion-weighted imaging modalities of MRI perfusion. This may have reduced the power of the study to inform clinical practice. Third, patients underwent only pre-interventional advanced perfusion imaging. Therefore, post-interventional reperfusion imaging was not available to compare infarct volume changes. To validate the differences between the one-stop ASL protocol and the one-stop pre-EVT protocol model, further multicenter randomized controlled trials with larger sample sizes and advanced equipment are warranted.

## 6. Conclusion

In patients with acute ischemic stroke within 24 h of onset, there were no significant differences in time efficiency and efficacy outcomes between the two perfusion groups. However, patients had lower sICH at 24 h and fewer EVT complications in the CTP group. Moreover, core infarct volume provided by advanced perfusion was an independent predictor of favorable outcome. Thus, perfusion images provide useful information for interventional decision-makers.

## Data availability statement

The raw data supporting the conclusions of this article will be made available by the authors, without undue reservation.

## Ethics statement

The studies involving humans were approved by the Medical Ethics Committee of the First Affiliated Hospital of Jinan University. The studies were conducted in accordance with the local legislation and institutional requirements. The participants provided their written informed consent to participate in this study.

## Author contributions

JG: Data curation, Investigation, Methodology, Software, Validation, Writing – original draft, Writing – review & editing. ZJ: Conceptualization, Investigation, Methodology, Visualization, Writing – original draft. SH: Data curation, Methodology, Investigation, Writing – review & editing. JY: Data curation, Methodology, Visualization, Writing – review & editing. MG: Conceptualization, Data curation, Visualization, Writing – review & editing. SZ: Conceptualization, Funding acquisition, Project administration, Resources, Writing – review & editing. HL: Data curation, Methodology, Project administration, Validation, Writing – review & editing. YL: Data curation, Methodology, Writing – review & editing. KL: Data curation, Methodology, Writing – review & editing. MY: Software, Visualization, Writing – review & editing. LH: Supervision, Writing – review & editing.
